# Benchmarking Prehospital and Emergency Department Care for Argentine Children with Traumatic Brain Injury: For the South American Guideline Adherence Group

**DOI:** 10.1371/journal.pone.0166478

**Published:** 2016-12-22

**Authors:** Monica S. Vavilala, Silvia B. Lujan, Qian Qiu, Gustavo J. Petroni, Nicolás M. Ballarini, Nahuel Guadagnoli, María Alejandra Depetris, Gabriela A. Faguaga, Gloria M. Baggio, Leonardo O. Busso, Mirta E. García, Osvaldo R. González Carrillo, Paula L. Medici, Silvia S. Sáenz, Elida E. Vanella, Anthony Fabio, Michael J. Bell

**Affiliations:** 1 Anesthesiology and Pain Medicine, Harborview Injury Prevention and Research Center, University of Washington, Seattle, Washington, United States of America; 2 Centro de Informática e Investigación Clínica, Rosario, Santa Fe, Argentina; 3 Hospital de emergencias Dr. Clemente Álvarez, Rosario, Santa Fe, Argentina; 4 Hospital de Niños Víctor J Vilela, Rosario, Santa Fe, Argentina; 5 Hospital El Cruce. Ezpeleta Oeste, Buenos Aires, Argentina; 6 Hospital de Niños Sor María Ludovica, La Plata, Buenos Aires, Argentina; 7 Hospital de Niños Dr. Orlando Alassia, Santa Fe, Santa Fe, Argentina; 8 Hospital Interzonal Especializado Materno Infantil Dr. Vitorio Tetamanti, Mar del Plata, Buenos Aires, Argentina; 9 Hospital de Niños de la Santísima Trinidad, Córdoba, Cordoba, Argentina; 10 Hospital Pediátrico Dr. Humberto Notti, Mendoza, Mendoza, Argentina; 11 Graduate School of Public Health, Epidemiology Data Coordinating Center, University of Pittsburgh, Pittsburgh, Pennsylvania, United States of America; 12 Neurological Surgery and Pediatrics, Critical Care Medicine, University of Pittsburgh, Pittsburgh, Pennsylvania, United States of America; Georgia Regents University Cancer Center, UNITED STATES

## Abstract

**Objective:**

There is little information on the type of early care provided to children with traumatic brain injury (TBI) in low middle income countries. We benchmarked early prehospital [PH] and emergency department [ED] pediatric TBI care in Argentina.

**Methods:**

We conducted a secondary analysis of data from patients previously enrolled in a prospective seven center study of children with TBI. Eligible participants were patients 0–18 years, and had diagnosis of TBI (admission Glasgow Coma scale score [GCS] < 13 or with GCS 14–15 and abnormal head CT scan within 48 hours of admission, and head AIS > 0). Outcomes were transport type, transport time, PH and ED adherence to best practice, and discharge Pediatric Cerebral Performance Category Scale (PCPC) and Pediatric Overall Performance category Scale (POPC).

**Results:**

Of the 366 children, mean age was 8.7 (5.0) years, 58% were male, 90% had isolated TBI and 45.4% were transported by private vehicle. 50 (34.7%) of the 144 children with severe TBI (39.3% of all TBI patients) were transported by private vehicle. Most (267; 73%) patients received initial TBI care at an index hospital prior to study center admission, including children with severe (81.9%) TBI. Transport times were shorter for those patients who were directly transported by ambulance to study center than for the whole cohort (1.4 vs.5.5 hours). Ambulance blood pressure data were recorded in 30.9%. ED guideline adherence rate was higher than PH guideline adherence rate (84.8% vs. 26.4%). For patients directly transferred from scene to study trauma centers, longer transport time was associated with worse discharge outcome (PCPC aOR 1.10 [1.04, 1.18] and (POPC aOR 1.10 [1.04, 1.18]). There was no relationship between PH or ED TBI guideline adherence rate and discharge POPC and PCPC.

**Conclusion:**

This study benchmarks early pediatric TBI care in Argentina and shows that many critically injured children with TBI do not receive timely or best practice PH care, that PH guideline adherence rate is low and that longer transport time was associated with poor discharge outcomes for patients with direct transfer status. There is an urgent need to improve the early care of children with TBI in Argentina, especially timely transportation to a hospital.

## Introduction

Injury related deaths worldwide have increased by 4.3 million deaths in 1990 to 4.8 million in 2013.[1,2] At-least 10 million worldwide, including children, are hospitalized or die from traumatic brain injury (TBI) annually.[3,4] Addressing this significant global health concern is a priority of the National Institutes of Health/ Fogarty International Center “Brain Disorders” program which mandates research on brain disorders, such as pediatric TBI, in low middle income countries (LMICs).

Unlike in the United States (U.S.) where the delivery of prehospital (PH) and emergency department (ED) care for TBI is supported by formal emergency medical systems which examine quality of care delivered to children with TBI, resource limited LMICs, struggle with poorly developed models of emergency medical services, trauma care access inequality, and few trained medical staff which limits their ability to benchmark PH and ED care.[5–7] This is problematic for LMICs because providing high quality PH and ED care in LMICs is necessary to achieve favorable pediatric TBI outcomes.[8]

Since examining the care of pediatric TBI patients requires using indicators that are relevant to local contexts is important, we previously tested the association between acute care indicators such as age, Glasgow Coma Scale score (GCS), hypotension, computed tomography (CT) findings, and pupillary reactivity that are commonly used in the developed world and evaluated the prognostic value of these variables in Argentina, and provided evidence that there is generalizability of the five World Health Organization/Organization Mondiale de la Santé TBI prognostic predictors in both high income countries and LMICs.[9–11] However, this study did not examine PH or ED care. In our recent multicenter U.S. based *Pediatric Guideline Adherence and Outcomes* study, we demonstrated that every 1 percentile increase in adherence to TBI Guideline indicators was associated with a 6% less in-patient mortality. [12, 13] We reported that direct transfer to a pediatric trauma center, and prevention and correction of hypoxia within 30 minutes after TBI in the pre-hospital and ED settings were protective. These findings and our prior work in Argentina, suggest an applicability of the U.S. based TBI Guidelines to countries like Argentina which do not have comparable prehospital infrastructure. To better understand early TBI care provided and in a LMIC setting, we leveraged our prior work and aimed to benchmark transport and the PH and ED management of children with TBI across multiple centers in Argentina.

## Materials and Methods

### Overview

This is an international collaborative study of seven Argentine pediatric trauma centers which formed a network to study TBI.[14] The present study is a secondary analysis of prospectively collected data from this network.[11] All seven study sites have Federal Wide Assurance approval. This study and the consent process was approved by the local ethical committees which were responsible for study oversight and the consent/assent process in Spanish. Hospitals and their respective IRB committees are: 1) Hospital de Niños Víctor J. Vilela, Rosario, Argentina (Secretaria Salud Pública Municipalidad de Rosario); 2) Hospital El Cruce, Florencio Varela, Argentina (Hospital Alta Complejidad El Cruce Dr. Nestor Carlos Kirchner); 3) SAMIC Hospital de Niños Sor María Ludovica, La Plata, Argentina (Hosp de Ninos de la Plata Sup Sor Maria Ludovica I RB; 4) Hospital de Niños “Dr. Orlando Alassia,” Santa Fe, Argentina (Comité de Etica en Investigación); 5) Hospital J. B. Iturraspe Hospital Interzonal Especializado Materno Infantil Dr. Vitorio Tetamanti, Mar del Plata, Argentina (Consejo Institucional de Revisión de Estudios de Investigación); 6) Hospital de Niños de la Santísima Trinidad, Córdoba, Argentina (Comité Insitucional de Etica de la Investigacion en salud del Niño y del Adulto); and 7) Polo Hospitalario Hospital Pediátrico Dr. Humberto Notti, Mendoza, Argentina (Hospital Central IRB).

The study and the consent process were approved by the local ethical committees which were responsible for study oversight and the consent/assent process in Spanish. Consent was obtained in writing from the next of kin, caretakers, or guardians on behalf of the minors/children enrolled in our study, and written child assent was attempted and obtained when possible for all children age 7 years and older). We documented consent processes and consents and stored consent in a secure file accessible only to investigators.

### Study Centers

All seven trauma hospitals can provide intracranial pressure (ICP) monitoring, have a TBI champion, and are public hospitals are located in large urban areas. Centers have computed tomography scanning capacity, an 8–24 bed pediatric intensive care unit, and round the clock neurosurgeon availability. Typically, TBI patients are evaluated in the ED and if needed, a head CT scan is performed within first 24 hours of injury. Teams for each study center included a principal investigator, community resource coordinator, data managers/translator, and data collectors.

### Data Sources and Data Collection

There were two data sources for this study: 1) the prospective study which evaluated the effectiveness and sustainability of two post TBI discharge trauma care protocols on 6 month functional outcomes among 308 children who survived to discharge with TBI [11] and 2) a dataset from these institutions of 58 patients who were part of another prospective observational study.

The instrument and data collection variables were developed according to the National Institute of Neurological Disorders and Stroke Common Data Elements project.[15] Data were collected on paper and then entered into a password protected web based database. Data cleaning was in real time by Argentine data managers. Site visits were conducted every 3 months to ensure data collection. For the present study, data were analyzed by NB, QQ and MSV. All patient evaluation, data collection and entry, quality control, and local administration were conducted by this group.

### Study Population

Eligible participants for this analysis were patients between 0–18 years of age admitted to one of the network EDs with a diagnosis of TBI (admission Glasgow Coma scale score [GCS] < 13 or with GCS 14–15 and abnormal head CT scan within 48 hours of admission, and head AIS > 0) and were followed up to hospital discharge. Recruitment ranged from August 2011 to June 2013. The severity of the TBI was determined by admission GCS (3–8: severe; 9–12: moderate; 13–15: mild). Participants who died in the ED were included. Severe TBI was defined by head AIS ≥ 3, abnormal head CT, and admission GCS < 9.

### Transport Characteristics

Transport type refers to how patients were transferred to from injury scene to first hospital (one of network hospitals or other hospitals) and were categorized by vehicle type and direct and indirect transfer status. Patients could be either transferred directly from scene to study hospitals (direct transfer), or transferred from index hospitals to study hospitals (indirect transfer). Transport time was calculated as the time difference (hours) from scene to study hospitals, and includes time spent at index hospitals.

### Measures of PH and ED Pediatric TBI Care

Five PH and six ED clinical indicators represented measures of adherence, in accordance with TBI Guidelines (Table 1) and scope of local practice. The number and type of indicators was determined *a priori* by the study group based on previous work and the feasibility of reliably interpreting these data collected as part of our prior work in Argentina. Some indicators were deemed to be conditional on occurrence and when denominator data were available, were examined as such.[13] Examples of conditional clinical indicators are treatment of hypoxia, and treatment of hypotension. Prehospital and ED indicators were abstracted form ED notes. Table 1 describes the list of PH and ED indicators used to determine TBI guideline adherence during PH and ED care. Choice of indicators to be included were adapted from evidence based recommendations [15] and adapted for the Argentine context by the Argentine study investigators, as deemed appropriate. Accordingly, we examined monitoring of blood pressure, receipt of intravenous fluids or colloids or blood products or vasopressors for hypotension, use of oxygen for hypoxia, and pupillary assessment. Hyperosmolar therapy use was examined among severe TBI patients; care that would otherwise be assumed to be standard of care in the developed world.

**Table 1:** Traumatic Brain Injury Care Indicators Evaluated for 2 Treatment locations (Pre-hospital (PH; 5) and Emergency Department (ED; 6).

Indicators	Definition and coding	PH*	ED**
		Indicator (n = 5)	Indicator (n = 6)
**1. Direct transfer from scene**	0 = No	X	
	1 = Yes		
	2 = Unknown		
**2. Blood pressure monitored**	0 = No	X	X
	1 = Yes		
	2 = Unknown		
**3. Hypotension treated** ***Condition*: *Hypotension***	0 = No		X
	1 = Yes		
	2 = No hypotension		
**4. Hypoxia monitored / Initial evaluation of oxygenation**	0 = No	X	X
	1 = Yes		
	2 = Unknown		
**5. Hypoxia treated** ***Condition*: *Hypoxia***	0 = No	X	X
	1 = Yes		
	2 = No hypoxia		
**6. Pupils assessed**	0 = No	X	X
	1 = Yes		
7. Hypertonic saline or mannitol used***	0 = No		X
	1 = Yes		

X –Indicator is applicable in that specific location

*Applicable to TBI patients transported by ambulance only (N = 110)

**Applicable to all TBI patients (N = 366)

***Applicable to severe TBI patients only (N = 144)

For each patient indicator, a value of 0 was assigned to lack of adherence and a value of 1 was assigned for adherence. When the adherence was unknown (i.e. unknown transfer status), a value of 2 was assigned, and these data were not included in calculation of patient and center level adherence rate. For conditional indicators such as treatment of hypoxia and hypotension, patients without hypoxia or hypotension were excluded from both numerator and denominator when calculating adherence rates.

The adherence rate for each patient at PH or ED was calculated using the sum of the numbers of indicators to which care was provided following TBI guidelines, divided by the sum of relevant indicators for that patient. Mean overall adherence rates at PH and ED for all patients at all study centers were further determined.

### Outcomes

Transport outcomes were transportation type and transportation time. Adherence outcomes were PH and ED guideline adherence rates. For transportation time and PH and ED guideline adherence rate effects [13], outcomes were discharge Pediatric Cerebral Performance Category Scale (PCPC) and Pediatric Overall Performance category Scale (POPC); [16] both are scored between one and six where score 1 = good, 2 = mild disability, 3 = moderate disability, 4 = severe disability, 5 = vegetative state, and 6 = death. A dichotomous measure of favorable (normal, mild-moderate disability) vs. poor outcome (severe-vegetative and death) was used for both PCPC and POPC.[16]

### Statistical Analyses

Patient demographic, clinical and transport details were described across seven centers. Data are presented as mean (standard errors) or median (interquartile range) for continuous variables and count (percentage) for categorical variables. Patient and injury level characteristics were examined in bivariate analyses by discharge PCPC and POPC, using Student’s t-test for continuous variables and χ2 tests for categorical variables. We determined PH care only for the 110 patients who were transported by ambulance. Adherence rate for each of five PH indicators and six ED indicators were compared across seven centers and mean PH and ED adherence rates were calculated for each center. We compared transport time from scene to study hospital for each trauma center and by direct transfer status.

Multivariate logistic regression analyses were used to examine the effect of transport time, and guideline adherence on PCPC and POPC at hospital discharge, stratified by transfer status. All multiple regressions adjusted for age, gender, maximum head AIS, maximum non-head AIS, and motor GCS at admission. All analyses controlled for clustering effect within each trauma center. Adjusted odds ratios and 95% CI were reported for transport time, and PH and ED adherence rate, which were used as continuous variables. Statistical significance was defined with a p value less than 0.05. Stata MP 13.1 (Stata Corporation, College Station, TX) was used for all analyses.

## Results

### Clinical Characteristics

Tables 2–4 show clinical data for the entire cohort and by each study center. Of the 366 children with TBI, patients were 8.7 (5.0) years, mostly (58.2%) male and 90% had isolated TBI. Traffic accidents exceeded falls by 10%, and over 93.2% of TBI was determined to be from accidental causes. The most common admission GCS motor score was 6 (42.9%) and the most common (30.6%) head abbreviated injury severity score was 3. One hundred and forty-four (39.3%) patients had severe TBI. Injury severity score was 15.1 (10.1). Cerebral contusions were the most common head CT diagnosis (26.2%) and 29.5% of TBI patients underwent surgery. Twenty three (6.3%) patients died and 21.6% had some form of discharge disability. Detailed clinical characteristics of the 366 children with TBI across seven study centers by discharge outcomes (univariate associations) is shown in Table S1 in S1 Table and Table S2 in S2 Table.

**Table 2:** Clinical Characteristics of 366* Children with Traumatic Brain Injury Admitted to Seven Study Centers.

	Total	*Center 1*	*Center 2*	*Center 3*	*Center 4*	*Center 5*	*Center 6*	*Center 7*
	*n = 366*	*n = 74*	*n = 95*	*n = 22*	*n = 80*	*n = 43*	*n = 25*	*n = 27*
	N(%)	*N(%)*	*N(%)*	*N(%)*	*N(%)*	*N(%)*	*N(%)*	*N(%)*
Age (years) mean[SD]	8.7[5.0]	8.4[4.5]	9.1[5.3]	10.7[3.8]	7.7[5.3]	9.9[5.8]	7.7[3.5]	7.9[4.1]
**Gender**								
Male	213 (58.2)	42 (56.8)	51 (53.7)	15 (68.2)	45 (56.3)	29 (67.4)	16 (64.0)	15 (55.6)
**Injury mechanism**								
Traffic accident	155 (42.4)	30 (40.5)	40 (42.1)	14 (63.6)	27 (33.8)	16 (37.2)	10 (40.0)	18 (66.7)
Fall from height	114 (31.2)	21 (28.4)	19 (20.0)	3 (13.6)	36 (45.0)	19 (44.2)	12 (48.0)	4 (14.8)
Fall from own height	19 (5.2)	4 (5.4)	7 (7.4)	1 (4.6)	3 (3.8)	2 (4.7)	0 (0.0)	2 (7.4)
Strike	41 (11.2)	11 (14.9)	13 (13.7)	1 (4.6)	10 (12.5)	2 (4.7)	1 (4.0)	3 (11.1)
Gunshot wound	11 (3.0)	2 (2.7)	5 (5.3)	2 (9.1)	0 (0.0)	2 (4.7)	0 (0.0)	0 (0.0)
Other / Unknown	26 (7.1)	6 (8.1)	11 (11.6)	1 (4.6)	4 (5.0)	2 (4.7)	2 (8.0)	0 (0.0)
**Injury circumstance**								
Child abuse	5 (1.4)	1 (1.4)	1 (1.1)	0 (0.0)	1 (1.3)	0 (0.0)	0 (0.0)	2 (7.4)
Intentional(no child abuse)	15 (4.1)	5 (6.8)	6 (6.3)	1 (4.6)	2 (2.5)	1 (2.3)	0 (0.0)	0 (0.0)
Accidental	341 (93.2)	67 (90.5)	85 (89.5)	21 (95.5)	76 (95.0)	42 (97.7)	25 (100.0)	25 (92.6)
Other / Unknown / Missing	5 (1.4)	1 (1.4)	3 (3.2)	0 (0.0)	1 (1.3)	0 (0.0)	0 (0.0)	0 (0.0)
**Glasgow coma scale score (admit motor)**								
1	78 (21.3)	9 (12.2)	27 (28.4)	5 (22.7)	16 (20.0)	11 (25.6)	7 (28.0)	3 (11.1)
2	10 (2.7)	1 (1.4)	4 (4.2)	3 (13.6)	1 (1.3)	0 (0.0)	0 (0.0)	1 (3.7)
3	5 (1.4)	1 (1.4)	1 (1.1)	2 (9.1)	1 (1.3)	0 (0.0)	0 (0.0)	0 (0.0)
4	26 (7.1)	3 (4.1)	3 (3.2)	6 (27.3)	4 (5.0)	2 (4.7)	2 (8.0)	6 (22.2)
5	28 (7.7)	3 (4.1)	11 (11.6)	1 (4.6)	5 (6.3)	0 (0.0)	0 (0.0)	8 (29.6)
6	157 (42.9)	38 (51.4)	36 (37.9)	0 (0.0)	49 (61.3)	23 (53.5)	3 (12.0)	8 (29.6)
Unknown	62 (16.9)	19 (25.7)	13 (13.7)	5 (22.7)	4 (5.0)	7 (16.3)	13 (52.0)	1 (3.7)
**Head abbreviated injury severity score (AIS)**								
1	9 (2.5)	0 (0.0)	1 (1.1)	0 (0.0)	0 (0.0)	0 (0.0)	8 (32.0)	0 (0.0)
2	70 (19.1)	16 (21.6)	15 (15.8)	4 (18.2)	16 (20.0)	15 (34.9)	3 (12.0)	1 (3.7)
3	112 (30.6)	27 (36.5)	33 (34.8)	5 (22.7)	16 (20.0)	17 (39.5)	4 (16.0)	10 (37.0)
4	100 (27.3)	14 (18.9)	26 (27.4)	5 (22.7)	32 (40.0)	7 (16.3)	7 (28.0)	9 (33.3)
5	54 (14.8)	14 (18.9)	10 (10.5)	7 (31.8)	14 (17.5)	2 (4.7)	3 (12.0)	4 (14.8)
6	21 (5.8)	3 (4.1)	10 (10.5)	1 (4.6)	2 (2.5)	2 (4.7)	0 (0.0)	3 (11.1)
**Injury severity score** mean[SD] **	15.1[10.1]	15.2[9.6]	15.2[10.3]	22.8[14.5]	15.1[10.0]	10.5[6.0]	13.0[9.5]	17.6[8.6]
**Non-head MAXAIS**								
0	229 (62.6)	48 (64.9)	53 (55.8)	7 (31.8)	69 (86.3)	32 (74.4)	8 (32.0)	12 (44.4)
1	41 (11.2)	11 (14.9)	11 (11.6)	3 (13.6)	3 (3.8)	0 (0.0)	9 (36.0)	4 (14.8)
2	34 (9.3)	3 (4.1)	11 (11.6)	3 (13.6)	4 (5.0)	7 (16.3)	3 (12.0)	3 (11.1)
3	32 (8.7)	6 (8.1)	8 (8.4)	6 (27.3)	3 (3.8)	2 (4.7)	4 (16.0)	3 (11.1)
4	5 (1.4)	1 (1.4)	0 (0.0)	1 (4.6)	0 (0.0)	0 (0.0)	1 (4.0)	2 (7.4)
5	4 (1.1)	2 (2.7)	1 (1.1)	1 (4.6)	0 (0.0)	0 (0.0)	0 (0.0)	0 (0.0)
6	1 (0.3)	0 (0.0)	1 (1.1)	0 (0.0)	0 (0.0)	0 (0.0)	0 (0.0)	0 (0.0)
Missing	20 (5.5)	3 (4.1)	10 (10.5)	1 (4.6)	1 (1.3)	2 (4.7)	0 (0.0)	3 (11.1)
Hospital stay (days)*** mean[SD]	11.3[14.1]	9.5[12.7]	12.2[16.3]	20.6[18.7]	9.2[10.6]	7.1[5.5]	19.1[22.2]	10.5[9.0]
**Extracranial injury**								
No	329 (90.0)	66 (89.2)	80 (84.2)	19 (86.4)	78 (97.5)	38 (88.4)	24 (96.0)	24 (88.9)
Yes	16 (4.4)	5 (6.8)	5 (5.3)	2 (9.1)	1 (1.3)	3 (7.0)	0 (0.0)	0 (0.0)
Missing	21 (5.7)	3 (4.1)	10 (10.5)	1 (4.6)	1 (1.3)	2 (4.7)	1 (4.0)	3 (11.1)
**Injury location**								
Head/Face	366 (100.0)	74 (100.0)	95 (100.0)	22 (100.0)	80 (100.0)	43 (100.0)	25 (100.0)	27 (100.0)
Neck	11 (3.0)	2 (2.7)	4 (4.2)	3 (13.6)	0 (0.0)	0 (0.0)	2 (8.0)	0 (0.0)
Thorax	48 (13.1)	12 (16.2)	14 (14.7)	8 (36.4)	4 (5.0)	5 (11.6)	4 (16.0)	1 (3.7)
Abdomen	34 (9.3)	8 (10.8)	8 (8.4)	5 (22.7)	3 (3.8)	2 (4.7)	6 (24.0)	2 (7.4)
Spine	9 (2.5)	1 (1.4)	3 (3.2)	3 (13.6)	0 (0.0)	0 (0.0)	1 (4.0)	1 (3.7)
Extremities	57 (15.6)	16 (21.6)	16 (16.8)	7 (31.8)	5 (6.3)	3 (7.0)	4 (16.0)	6 (22.2)
External and Other	12 (3.3)	1 (1.4)	5 (5.3)	2 (9.1)	1 (1.3)	0 (0.0)	1 (4.0)	2 (7.4)
**All head computed tomography diagnoses**								
Epidural hematoma	5 (1.4)	0 (0.0)	1 (1.1)	0 (0.0)	1 (1.3)	1 (2.3)	1 (4.0)	1 (3.7)
Subdural hematoma	40 (10.9)	13 (17.6)	4 (4.2)	5 (22.7)	6 (7.5)	4 (9.3)	5 (20.0)	3 (11.1)
Subarachnoid hemorrhage	25 (6.8)	7 (9.5)	6 (6.3)	0 (0.0)	9 (11.3)	0 (0.0)	0 (0.0)	3 (11.1)
Intracerebral hemorrhage	11 (3.0)	1 (1.4)	0 (0.0)	2 (9.1)	3 (3.8)	2 (4.7)	2 (8.0)	1 (3.7)
Intraventricular hemorrhage	16 (4.4)	3 (4.1)	5 (5.3)	0 (0.0)	5 (6.3)	2 (4.7)	0 (0.0)	1 (3.7)
Contusion	96 (26.2)	32 (43.2)	25 (26.3)	5 (22.7)	13 (16.3)	3 (7.0)	10 (40.0)	8 (29.6)
**Any surgery**								
No	258 (70.5)	56 (75.7)	71 (74.7)	17 (77.3)	50 (62.5)	25 (58.1)	16 (64.0)	23 (85.2)
Yes	108 (29.5)	18 (24.3)	24 (25.3)	5 (22.7)	30 (37.5)	18 (41.9)	9 (36.0)	4 (14.8)
**Decompressive craniectomy**								
No	344 (94.0)	72 (97.3)	91 (95.8)	16 (72.7)	75 (93.8)	42 (97.7)	21 (84.0)	27 (100.0)
Yes	19 (5.2)	2 (2.7)	4 (4.2)	6 (27.3)	5 (6.3)	0 (0.0)	2 (8.0)	0 (0.0)
NA / Missing	3 (0.8)	0 (0.0)	0 (0.0)	0 (0.0)	0 (0.0)	1 (2.3)	2 (8.0)	0 (0.0)

*Among 366 study sample, 39% (N = 144) were identified as severe TBI patients (head AIS ≥ 3, abnormal head CT); 5% (N = 20) died at admission.

**Among 366 study sample, 20 patients died at admission had missing injury severity score (Center1 [N = 71]; Center2 [N = 85]; Center3 [N = 21]; Center4 [N = 79]; Center5 [N = 41]; Center6 [N = 25]; Center7 [N = 24])

***Among 366 study sample, 20 patients died at admission (their hospital LOS was assumed to be 1 day), and LOS could not be calculated for 1 patient due to missing discharge date (Center1 [N = 74]; Center2 [N = 94]; Center3 [N = 22]; Center4 [N = 80]; Center5 [N = 43]; Center6 [N = 25]; Center7 [N = 27])

**Table 3:** Transfer and Transport Characteristics of 366* Children with Traumatic Brain Injury Admitted to Seven Study Centers.

	Total	*Center 1*	*Center 2*	*Center 3*	*Center 4*	*Center 5*	*Center 6*	*Center 7*
	*n = 366*	*n = 74*	*n = 95*	*n = 22*	*n = 80*	*n = 43*	*n = 25*	*n = 27*
	N(%)	*N(%)*	*N(%)*	*N(%)*	*N(%)*	*N(%)*	*N(%)*	*N(%)*
Transportation type**								
Private vehicle	166 (45.4)	33 (44.6)	41 (43.2)	3 (13.6)	54 (67.5)	15 (34.9)	13 (52.0)	7 (25.9)
Helicopter	5 (1.4)	0 (0.0)	1 (1.1)	0 (0.0)	2 (2.5)	1 (2.3)	0 (0.0)	1 (3.7)
Ambulance	110 (30.1)	27 (36.5)	25 (26.3)	14 (63.6)	17 (21.3)	4 (9.3)	8 (32.0)	15 (55.6)
Other/Unknown	85 (23.2)	14 (18.9)	28 (29.5)	5 (22.7)	7 (8.8)	23 (53.5)	4 (16.0)	4 (14.8)
**Direct transfer from scene**								
Yes	96 (26.2)	24 (32.4)	22 (23.2)	6 (27.3)	1 (1.3)	19 (44.2)	9 (36.0)	15 (55.6)
No	267 (73.0)	50 (67.6)	72 (75.8)	16 (72.7)	78 (97.5)	23 (53.5)	16 (64.0)	12 (44.4)
Unknown	3 (0.8)	0 (0.0)	1 (1.1)	0 (0.0)	1 (1.3)	1 (2.3)	0 (0.0)	0 (0.0)
**Transport time from scene to study hospitals (hours)** ***								
mean[SD]	5.5[6.3]	4.6[6.9]	6.7[7.1]	5.1[6.7]	7.2[6.0]	4.4[4.9]	5.0[5.4]	2.1[1.6]
Median[IQR]	3.7[4.2]	2.5[4.2]	4.6[5.3]	3.5[4.9]	4.9[5.9]	3.0[2.8]	3.5[3.3]	1.8[2.1]

*Among 366 study sample, 39% (N = 144) were identified as severe TBI patients (head AIS ≥ 3, abnormal head CT); 5% (N = 20) died at admission.

**Transportation type has four categories. Private Vehicle combines “Private vehicle” and “Taxi”; Helicopter includes “Helicopter” only; Ambulance combines “Ambulance (doctor)”, “Police”, and “Firefighter’; Other/Unknown combines “Other” and “Unknown

*** Among 366 study sample, 27 patients had missing transport time (Center1 [N = 70]; Center2 [N = 80]; Center3 [N = 19]; Center4 [N = 79]; Center5 [N = 41]; Center6 [N = 24]; Center7 [N = 26])

**Table 4:** Outcome Characteristics of 366* Children with Traumatic Brain Injury Admitted to Seven Study Centers.

	Total	*Center 1*	*Center 2*	*Center 3*	*Center 4*	*Center 5*	*Center 6*	*Center 7*
	*n = 366*	*n = 74*	*n = 95*	*n = 22*	*n = 80*	*n = 43*	*n = 25*	*n = 27*
	N(%)	*N(%)*	*N(%)*	*N(%)*	*N(%)*	*N(%)*	*N(%)*	*N(%)*
**Discharge disposition**								
Home w/o homecare	330 (90.2)	66 (89.2)	82 (86.3)	20 (90.9)	72 (90.0)	41 (95.4)	25 (100.0)	24 (88.9)
Home with homecare	4 (1.1)	3 (4.1)	0 (0.0)	0 (0.0)	1 (1.3)	0 (0.0)	0 (0.0)	0 (0.0)
Other hospital	7 (1.9)	0 (0.0)	2 (2.1)	1 (4.6)	4 (5.0)	0 (0.0)	0 (0.0)	0 (0.0)
Other	2 (0.6)	2 (2.7)	0 (0.0)	0 (0.0)	0 (0.0)	0 (0.0)	0 (0.0)	0 (0.0)
Death	23 (6.3)	3 (4.1)	11 (11.6)	1 (4.6)	3 (3.8)	2 (4.7)	0 (0.0)	3 (11.1)
**Pediatric Cerebral Performance category Scale (PCPC) at Hospital discharge**								
Normal	264 (72.1)	56 (75.7)	65 (68.4)	10 (45.5)	62 (77.5)	37 (86.1)	15 (60.0)	19 (70.4)
Mild disability	38 (10.4)	9 (12.2)	8 (8.4)	4 (18.2)	4 (5.0)	4 (9.3)	6 (24.0)	3 (11.1)
Moderate disability	15 (4.1)	2 (2.7)	9 (9.5)	1 (4.6)	2 (2.5)	0 (0.0)	0 (0.0)	1 (3.7)
Severe disability	23 (6.3)	3 (4.1)	2 (2.1)	4 (18.2)	9 (11.3)	0 (0.0)	4 (16.0)	1 (3.7)
Coma or vegetative state	3 (0.8)	1 (1.4)	0 (0.0)	2 (9.1)	0 (0.0)	0 (0.0)	0 (0.0)	0 (0.0)
Brain death	23 (6.3)	3 (4.1)	11 (11.6)	1 (4.6)	3 (3.8)	2 (4.7)	0 (0.0)	3 (11.1)
**Pediatric Overall Performance Category Scale (POPC) at Hospital discharge**								
Good overall performance	263 (71.9)	59 (79.7)	61 (64.2)	9 (40.9)	61 (76.3)	37 (86.1)	17 (68.0)	19 (70.4)
Mild overall performance	36 (9.8)	5 (6.8)	11 (11.6)	4 (18.2)	5 (6.3)	4 (9.3)	4 (160.)	3 (11.1)
Moderate overall performance	20 (5.5)	4 (5.4)	9 (9.5)	4 (18.2)	2 (2.5)	0 (0.0)	0 (0.0)	1 (3.7)
Severe overall performance	21 (5.7)	2 (2.7)	3 (3.2)	2 (9.1)	9 (11.3)	0 (0.0)	4 (16.0)	1 (3.7)
Coma or vegetative state	3 (0.8)	1 (1.4)	0 (0.0)	2 (9.1)	0 (0.0)	0 (0.0)	0 (0.0)	0 (0.0)
Death	23 (6.3)	3 (4.1)	11 (11.6)	1 (4.6)	3 (3.8)	2 (4.7)	0 (0.0)	3 (11.1)

*Among 366 study sample, 39% (N = 144) were identified as severe TBI patients (head AIS ≥ 3, abnormal head CT); 5% (N = 20) died at admission.

### Transfer Status

Most (267; 73%) patients received initial TBI care at an index hospital prior to transfer to a study centers (Table 2).

### Transport Type

The most common (45.4%) mode of transportation to the study hospital was by private vehicle, followed by ambulance (30.1%). Fifty (34.7%) of the 144 children with severe TBI (39.3% of all TBI patients) were transported by private vehicle. Of the 166 patients transported by private vehicle, 30.1% had with severe TBI and of the 110 patients transported by ambulance, 51.8% were had severe TBI. A smaller proportion of severe (18.1%) TBI patients were directly transferred compared to moderate (32.3%) and mild TBI patients (31.9%; p = 0.01).

### Transport Times

Transport times from scene to non-study center index hospital were available for 109 of 267 (41%) patients whereas transport time was also available for 339 (92.6%) patients transported from scene to study center. Three hundred and thirty six patients had complete data on both transport time and transfer status data from scene to study center.

**Fig 1:**
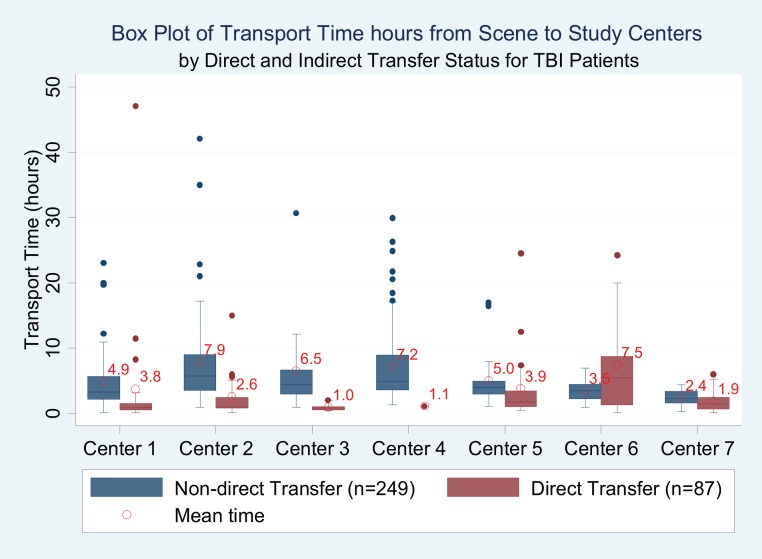
Box Plot of Transport Time hours from Scene to Study Centers by Direct and Indirect Transfer Status for TBI Patients. * Among 366 study sample, 27 patients had missing transport time, and 3 patients had unknown direct transfer status (Center1 [N = 70]; Center2 [N = 79]; Center3 [N = 19]; Center4 [N = 78]; Center5 [N = 40]; Center6 [N = 24]; Center7 [N = 26]).

**Fig 2:**
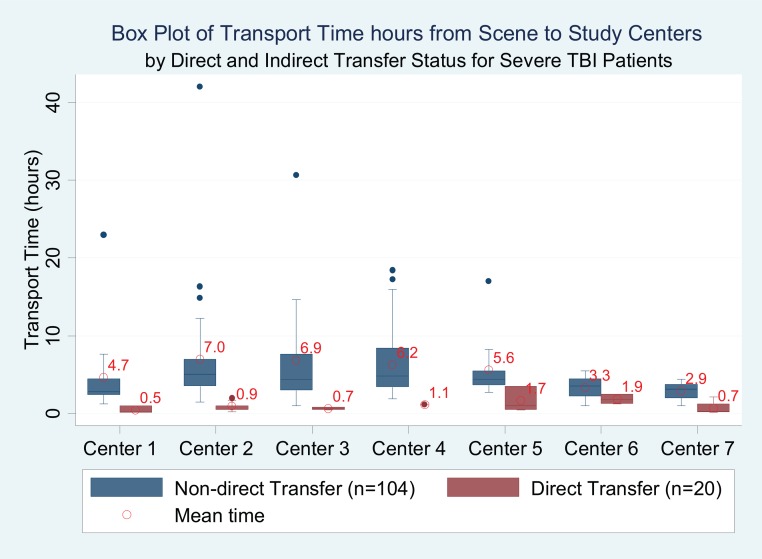
Box Plot of Transport Time hours from Scene to Study Centers by Direct and Indirect Transfer Status for Severe TBI Patients. * Among 144 study sample with severe TBI, 20 patients had missing transport time (Center1 [N = 16]; Center2 [N = 35]; Center3 [N = 14]; Center4 [N = 27]; Center5 [N = 13]; Center6 [N = 9]; Center7 [N = 10]).

For all 366 patients, including direct and those who received initial care at an index hospital and those who arrived by private vehicle, mean transport time from scene to study center was 5.5 hours (6.3; range 2.1–7.2 hours). For the 37.5% of patients who were transported directly from scene to study centers by ambulance, the average transport time from scene to study center was 1.4 (1.4) hours. Seven three patients who were transported by ambulance to an index hospital, received initial care, and then transferred to study centers had an average transport time from scene to index hospital of 1.2 (SD 1.1) hours; no different from those patients who were transferred directly from scene to the study center. There was less center variation in transport time for the cohort of severe TBI, as compared to the entire TBI cohort (Figs 1 and 2, example Center 6).

### Transport Time and Outcomes

While 21.6% of survivors discharged home had some degree of disability, over 90% of children with TBI were discharged home without health care. There were 336 (92%) children with complete data on transport time, transfer status and outcomes (POPC and PCPC). For patients directly transferred from scene, longer transport time was associated with worse discharge outcome (PCPC aOR 1.10 [1.04, 1.18] and (POPC aOR 1.10 [1.04, 1.18]) (Table 5).

**Table 5:** Association between Transport Time (hours) from Scene to Study Centers (Including Transfers) and Discharge Outcomes in 366* Children with Traumatic Brain Injury.

	Direct Transfer from Scene (n = 87)		Indirect Transfer (n = 249)	
	Poor PCPC**	Poor POPC***	Poor PCPC**	Poor POPC***
Variable	aOR (95% CI)	aOR (95% CI)	aOR (95% CI)	aOR (95% CI)
Transport time from scene to study hospitals (hours)	1.10 (1.04, 1.18)	1.10 (1.04, 1.18)	0.95 (0.86, 1.04)	0.95 (0.88, 1.03)

* Among 366 study sample, 27 patients had missing transport time, and 3 patients had unknown direct transfer status. Risk estimates are adjusted odds ratios (ORs) of transport time for PCPC and POPC, and adjusted for age, gender, maximum head Abbreviated Injury Score (AIS), maximum non-head AIS and Glasgow Coma Scale score motor

**Dichotomous PCPC (favorable outcome = normal, mild-moderate disability vs. poor outcome = severe-vegetative and death)

***Dichotomous POPC (favorable outcome = good-moderate overall performance vs. poor outcome = severe-vegetative state and death)

### Measures of PH and ED TBI Care

Prehospital care was benchmarked for the 110 patients with TBI transported by ambulance. Prehospital guideline adherence was 24.6% with large center variation (Table 6). Blood pressure was recorded in only 30.9% of patients and there was record of only 6.4% of those with hypotension receiving treatment. Thirty three children (30%) underwent oxygenation monitoring; 8.2% of these patients had hypoxia with over 80% successfully treated.

**Table 6:** Traumatic Brain Injury Clinical Care Indicators Evaluated Across Seven Study Centers by Pre-hospital [PH] Care of Patients Transported by Ambulance (N = 110), and in Emergency Department (ED; N = 366).

***PH***	**Indicator**	***Total***	***Center 1***	***Center 2***	***Center 3***	***Center 4***	***Center 5***	***Center 6***	***Center 7***
		***(n = 110)***	***(n = 27)***	***(n = 25)***	***(n = 14)***	***(n = 17)***	***(n = 4)***	***(n = 8)***	***(n = 15)***
		***%***	***%***	***%***	***%***	***%***	***%***	***%***	***%***
Indicator 1	Direct transfer from scene*	33.0	40.7	29.2	35.7	0.0	50.0	25.0	60.0
Indicator 2	Blood pressure monitored (n = 34)	30.9	37.0	12.0	28.6	35.3	0.0	37.5	53.3
	% patients with hypotension (n = 7)	6.4	11.1	4.0	21.4	0.0	0.0	0.0	0.0
Indicator 4	Hypoxia monitored/initial oxygen evaluation (n = 33)	30.0	37.0	12.0	21.4	35.3	0.0	37.5	53.3
	% patients with hypoxia (n = 9)	8.2	14.8	4.0	14.3	0.0	0.0	12.5	6.7
Indicator 5	Hypoxia treated among those with hypoxia**(n = 5)	83.3	75.0	100.0	100.0	---	---	---	---
Indicator 6	Pupils assessed	3.6	11.1	0.0	7.1	0.0	0.0	0.0	0.0
***PH Adherence rate (%)***		***24*.*6***	***32*.*0***	***13*.*2***	***23*.*9***	***17*.*6***	***12*.*5***	***25*.*0***	***41*.*7***
**ED**	**Indicator**	***Total***	***Center 1***	***Center 2***	***Center 3***	***Center 4***	***Center 5***	***Center 6***	***Center 7***
		***(n = 366)***	***(n = 74)***	***(n = 95)***	***(n = 22)***	***(n = 80)***	***(n = 43)***	***(n = 25)***	***(n = 27)***
		***%***	***%***	***%***	***%***	***%***	***%***	***%***	***%***
Indicator 2	Blood pressure monitored (n = 346)	94.5	85.1	96.8	100.0	100.0	90.7	96.0	96.3
	% patients with hypotension (n = 36)	9.8	9.5	12.6	31.8	8.8	0.0	12.0	0.0
Indicator 3	Hypotension treated among those with hypotension*** (n = 28)	93.3	100.0	87.5	100.0	85.7	---	100.0	---
Indicator 4	Hypoxia monitored/initial oxygen evaluation (n = 350)	95.6	85.1	99.0	100.0	100.0	97.7	100.0	88.9
	% patients with hypoxia (n = 18)	4.9	5.4	7.4	13.6	2.5	0.0	4.0	3.7
Indicator 5	Hypoxia treated among those with hypoxia****(n = 10)	83.3	75.0	100.0	100.0	50.0	---	100.0	---
Indicator 6	Pupils assessed	83.3	81.1	94.7	72.7	93.8	90.7	20.0	74.1
Indicator 7	Hypertonic saline used among severe TBI (N = 124 denominator)*****	17.0	26.7	16.7	25.0	14.8	7.7	0.0	28.6
***Mean ED Adherence rate (%)***		***84*.*8***	***80*.*2***	***89*.*1***	***80*.*1***	***90*.*7***	***86*.*4***	***67*.*1***	***82*.*4***

Among the cohort of 366 patients, 20 died at admission and 346 were followed up to hospital discharge. Data includes the 6 patients who were transported by ambulance and died in the ED. Results did not change with removal of data from these 6 children.

PH data exclude patients brought to hospitals by private cars even though 30% of patients with severe TBI were transported by private vehicles (no monitoring or treatment capacity).

* 1 patients with unknown direct transfer status were not included in adherence calculation (Center1 [N = 27]; Center2 [N = 24]; Center3 [N = 14]; Center4 [N = 17]; Center5 [N = 4]; Center6 [N = 8]; Center7 [N = 15])

** PH hypoxia treated is a conditional indicator. Three patients with hypoxia but with unknown treatment information were not included in adherence calculation (Center1 [N = 3]; Center2 [N = 0]; Center3 [N = 1]; Center4 [N = 0]; Center5 [N = 0]; Center6 [N = 0]; Center7 [N = 0])

*** ED hypotension treated is a conditional indicator (Center1 [N = 5]; Center2 [N = 8]; Center3 [N = 7]; Center4 [N = 7]; Center5 [N = 0]; Center6 [N = 3]; Center7 [N = 0])

**** ED hypoxia treated is a conditional indicator (Center1 [N = 4]; Center2 [N = 2]; Center3 [N = 3]; Center4 [N = 2]; Center5 [N = 0]; Center6 [N = 1]; Center7 [N = 0])

*****Applicable to severe TBI patients with completed data only (Center1 [N = 15]; Center2 [N = 36]; Center3 [N = 16]; Center4 [N = 27]; Center5 [N = 13]; Center6 [N = 10]; Center7 [N = 7])

---Means the data is not applicable or missing or unknown

Emergency department care was benchmarked for all 366 children with TBI. Overall ED guideline adherence was 84.8%. Three hundred and forty-six (94.5%) patients had blood pressure recorded, 9.8% of these patients had hypotension. Hypotension was successfully treated in over 90%. Over 95% of children had oxygenation monitored; 18 (4.9%) of those monitored had hypoxia and over 80% of these patients were successfully treated. Less than 1/3 of children with severe TBI received hypertonic saline. There was no relationship between PH or ED TBI Guideline adherence rate and discharge outcomes (Table 7).

**Table 7:** Association between PH and ED Guideline Adherence and Discharge Outcomes in 366* Children with Traumatic Brain Injury.

	PH (n = 110)		ED (n = 346)	
	Poor PCPC**	Poor POPC***	Poor PCPC**	Poor POPC***
Variable	aOR (95% CI)	aOR (95% CI)	aOR (95% CI)	aOR (95% CI)
Guideline adherence rate	1.00 (0.99, 1.02)	0.99 (0.97, 1.01)	1.03 (0.99, 1.06)	1.03 (0.99, 1.06)

*Among the cohort of 366 patients, 20 died at admission and 346 were followed up to hospital discharge. PH adherence analysis was performed for 110 patients transported by ambulance. 20 patients died at admission were not included in ED adherence analysis. Risk estimates are adjusted odds ratios (ORs) of adherence rate for PCPC and POPC, and adjusted for age, gender, maximum head Abbreviated Injury Score (AIS), maximum non-head AIS and Glasgow Coma Scale score motor

**Dichotomous PCPC (favorable outcome = normal, mild-moderate disability vs. poor outcome = severe-vegetative and death)

***Dichotomous POPC (favorable outcome = good-moderate overall performance vs. poor outcome = severe-vegetative state and death)

## Discussion

In this study, we found that: 1) Transport times to tertiary pediatric trauma centers exceed 5 hours, 2) Longer transport times are associated with worse discharge outcomes, 3) Few patients receive PH ambulance care and PH guideline adherence rate is low for those who receive ambulance care, and 4) There was no relationship between guideline adherence rate and discharge PCPC/POPC. This is the first and largest study to examine and benchmark PH and ED pediatric TBI care in South America and the largest study of isolated TBI in children from an LMIC.

Access to early trauma care is a high priority global health quality improvement concern where lack of ambulances, lack of high quality PH care, and lack of trauma systems are recognized to be significant barriers to providing best practice TBI care. A 2015 review of studies of trauma systems identified only 10 unique LMIC EMS and trauma system programs where initiatives included the integration of existing PH and trauma services, provision of standardized training and formalized certification processes for PH providers, as well as the construction of a public health based conceptual framework trauma care.[6] Another study used cost distance methods to demonstrate the effectiveness and value of increased national ambulance service in reducing access inequality in rural and urban Ghana.[7] In this study, we did not have ambulance station data but were able to document transport times and the proportion of critically injured Argentine children who would be in need of timely ambulance services. Our findings that so many children with severe TBI are transported by private vehicle coupled with a low PH guideline adherence rate for those who were transported by ambulance are alarming given the urgent need of expedient vascular access and resuscitation treatments that these patients require.[17] While it is possible that critically injured children received better ambulance care than reported in this study, there are many children with severe TBI who are transported by private vehicle who do not have the opportunity to receive any early care. For more severely injured patients, the delay of a 5 hour transport time is well to develop EMS infrastructure, capacity building and PH educational strategies to provide high quality early pediatric TBI care are urgently needed.

For patients directly transferred from scene to study trauma centers, our data show that shorter transport times was associated with better discharge outcomes. For patients with indirect transfer status, transport time was not associated with outcome. Two factors may explain differences in the transport time effect. First, many patients received initial care elsewhere before transfer to a study hospital which may have resulted in successful resuscitation and stabilization[18]. Second, patients who arrived at an index hospital where they may have received TBI care and then transported to one of the trauma centers; in this case, transport time may be less important than timely quality care.

There was no relationship between guideline adherence and PCPC and POPC, which may be due to our adaptation of the guideline indicators to be more process measures and more surrogate than in the BTF Guidelines.[13] For example, even if blood pressure was monitored, documentation of PH resuscitation and basic vital sign monitoring was low, and if transport time was very long, then ED guideline adherence may not influence outcomes as it would in the U.S. In this cohort, twenty patients died at or even prior to ED admission were not included in ED adherence and outcome analysis since they would not have the opportunity to receive guideline adherence.

Engaging the public sector on traffic and the built environment, and use of audit filters, registries, preventable death panels, and morbidity and mortality conferences, as recommended by the WHO Guidelines for trauma quality improvement program, are opportunities to reduce PH transport time, improve PH care, and to improve the PH care of children with TBI.[13] Since the study centers do not have standardized quality improvement programs, we are not able to examine quality improvement program elements at each center.[6, 19–24] Although PH or ED guideline adherence rates were not associated with discharge outcomes, TBI guideline adherence can be used as a measure of quality of pediatric TBI care in LMICs and may be a measure to evaluate best practice TBI processes of care.

Although not the focus of this study, we observed that although over 90% of survivors were discharged home without healthcare and slightly over 1% was discharged home with some form of health services, over 20% had mild, moderate or severe disability at hospital discharge. This observation is similar to that observed by Gupta and colleagues in adult TBI in the Indian context where the lack of follow up and rehabilitation is associated with poor post discharge trajectories despite high quality in-hospital TBI care.[25, 26] Yet, the recently published study of 6 month outcomes in a similar cohort reported no effect on a community resource on 6 month outcomes, [11] suggesting that high quality early TBI care may also be needed to realize the benefits of downstream community or rehabilitation services that facilitate achieve good long term outcomes.

This study has some limitations. First, we do not have data for this entire cohort beyond hospital discharge. Second, clinical care information from transferring hospitals was not collected as part of the parent study, thereby not allowing us to fully evaluate all aspects of care after the child was injured. We did not gather information on TBI education and cannot assess the role of PH and ED provider knowledge on TBI care or the number of reasons why children with TBI were transported by private vehicle rather than by ambulance. Most children were transported by private vehicle whose condition during transport could not be examined and PH documentation of care was poor. Finally, we do not know how many children with TBI died at the scene and or transported to other treatment facilities not included in this network. However, while not a population based study of the epidemiology of TBI in Argentina, and despite the aforementioned limitations, the present study provides new information on the current state of acute pediatric TBI care in Argentina.

In summary, there is an urgent need to improve early pediatric TBI care in South America. This study is the first and largest study to benchmark the early care of children with TBI in Argentina. Findings are that many critically injured children with TBI do not receive any PH care, transport times are unacceptably long and harmful, and that ambulance PH guideline adherence rate is alarmingly low.

## Supporting Information

S1 Table Clinical Characteristics of 366 Children with Trauma Brain Injury across Seven Study Centers by Discharge Outcomes (Univariate Associations). p values are corrected by adjusting clustering effect within trauma centers
*Dichotomous PCPC (favorable outcome = normal, mild-moderate disability vs. poor outcome = severe-vegetative and death)
**Dichotomous POPC (favorable outcome = good-moderate overall performance vs. poor outcome = severe-vegetative state and death)
*****Among 366 study sample, 20 patients died at admission had missing injury severity score (Center1 [N = 71]; Center2 [N = 85]; Center3 [N = 21]; Center4 [N = 79]; Center5 [N = 41]; Center6 [N = 25]; Center7 [N = 24])
*****Among 366 study sample, 20 patients died at admission (their hospital LOS was assumed to be 1 day), and LOS could not be calculated for 1 patient due to missing discharge date (Center1 [N = 74]; Center2 [N = 94]; Center3 [N = 22]; Center4 [N = 80]; Center5 [N = 43]; Center6 [N = 25]; Center7 [N = 27])
(DOCX)

S2 Table Transportation Characteristics of 366 Children with Trauma Brain Injury across Seven Study Centers by Discharge Outcomes (Univariate Associations). p values are corrected by adjusting clustering effect within trauma centers
*Dichotomous PCPC (favorable outcome = normal, mild-moderate disability vs. poor outcome = severe-vegetative and death)
**Dichotomous POPC (favorable outcome = good-moderate overall performance vs. poor outcome = severe-vegetative state and death)
***Transportation type has four categories. Private Vehicle combines “Private vehicle” and “Taxi”; Helicopter includes “Helicopter” only; Ambulance combines “Ambulance (doctor)”, “Police”, and “Firefighter’; Other/Unknown combines “Other” and “Unknown
**** Among 366 study sample, 27 patients had missing transport time (Center1 [N = 70]; Center2 [N = 80]; Center3 [N = 19]; Center4 [N = 79]; Center5 [N = 41]; Center6 [N = 24]; Center7 [N = 26])
(DOCX)
