# Supplementary material for: Benchmarking Prehospital and Emergency Department Care for Argentine Children with Traumatic Brain Injury: For the South American Guideline Adherence Group
Source: PLoS One. 2016 Dec 22;11(12):e0166478. doi: 10.1371/journal.pone.0166478 (PMC5179077; doi:10.1371/journal.pone.0166478)
Supplement: S1 Table — p values are corrected by adjusting clustering effect within trauma centers *Dichotomous PCPC (favorable outcome = normal, mild-moderate disability vs. poor outcome = severe-vegetative and death) **Dichotomous POPC (favorable outcome = good-moderate overall performance vs. poor outcome = severe-vegetative state and death) *****Among 366 study sample, 20 patients died at admission had missing injury severity score (Center1 [N = 71]; Center2 [N = 85]; Center3 [N = 21]; Center4 [N = 79]; Center5 [N = 41]; Center6 [N = 25]; Center7 [N = 24]) *****Among 366 study sample, 20 patients died at admission (their hospital LOS was assumed to be 1 day), and LOS could not be calculated for 1 patient due to missing discharge date (Center1 [N = 74]; Center2 [N = 94]; Center3 [N = 22]; Center4 [N = 80]; Center5 [N = 43]; Center6 [N = 25]; Center7 [N = 27]) (DOCX) [file pone.0166478.s001.docx]

**S1 Table: Clinical Characteristics of 366 Children with Trauma Brain Injury across Seven Study Centers by Discharge Outcomes (Univariate Associations).**

|  | **Total** | ***Favorable PCPC**** | ***Poor PCPC**** | ***P-value*** | ***Favorable POPC***** | ***Poor POPC***** | ***P-value*** |
| --- | --- | --- | --- | --- | --- | --- | --- |
|  | ***n=366*** | ***n=317*** | ***n=49*** |  | ***n=319*** | ***n=47*** |  |
|  | **N(%)** | ***N(%)*** | ***N(%)*** |  | ***N(%)*** | ***N(%)*** |  |
| Age (years) mean[SD] | 8.7[5.0] | 8.7[5.0] | 8.6[4.7] | 0.95 | 8.7[5.0] | 8.6[4.8] | 0.93 |
|  |  |  |  |  |  |  |  |
| **Gender** |  |  |  |  |  |  |  |
| Male | 213 (58.2) | 183 (57.7) | 30 (61.2) | 0.69 | 185 (58.0) | 28 (59.6) | 0.84 |
|  |  |  |  |  |  |  |  |
| **Injury mechanism** |  |  |  | **0.02** |  |  | **0.02** |
| Traffic accident | 155 (42.4) | 124 (39.1) | 31 (63.3) |  | 126 (39.5) | 29 (61.7) |  |
| Fall from height | 114 (31.2) | 106 (33.4) | 8 (16.3) |  | 106 (33.2) | 8 (17.0) |  |
| Fall from own height | 19 (5.2) | 19 (6.0) | 0 (0.0) |  | 19 (6.0) | 0 (0.0) |  |
| Strike | 41 (11.2) | 39 (12.3) | 2 (4.1) |  | 39 (12.2) | 2 (4.3) |  |
| Gunshot wound | 11 (3.0) | 6 (1.9) | 5 (10.2) |  | 6 (1.9) | 5 (10.6) |  |
| Other / Unknown | 26 (7.1) | 23 (7.3) | 3 (6.1) |  | 23 (7.2) | 3 (6.4) |  |
|  |  |  |  |  |  |  |  |
| **Injury circumstance** |  |  |  | **0.006** |  |  | **0.003** |
| Child abuse | 5 (1.4) | 2 (0.6) | 3 (6.1) |  | 2 (0.6) | 6 (6.4) |  |
| Intentional(no child abuse) | 15 (4.1) | 13 (4.1) | 2 (4.1) |  | 13 (4.1) | 2 (4.3) |  |
| Accidental | 341 (93.2) | 299 (94.3) | 42 (85.7) |  | 301 (94.4) | 40 (85.1) |  |
| Other / Unknown / Missing | 5 (1.4) | 3 (1.0) | 2 (4.1) |  | 3 (0.9) | 2 (4.3) |  |
|  |  |  |  |  |  |  |  |
| **Glasgow coma scale score (admit motor)** |  |  |  | **0.0002** |  |  | **0.0002** |
| 1 | 78 (21.3) | 54 (17.0) | 24 (49.0) |  | 55 (17.2) | 23 (48.9) |  |
| 2 | 10 (2.7) | 4 (1.3) | 6 (12.2) |  | 4 (1.3) | 6 (12.8) |  |
| 3 | 5 (1.4) | 3 (1.0) | 2 (4.1) |  | 3 (0.9) | 2 (4.3) |  |
| 4 | 26 (7.1) | 22 (6.9) | 4 (8.2) |  | 22 (6.9) | 4 (8.5) |  |
| 5 | 28 (7.7) | 26 (8.2) | 2 (4.1) |  | 26 (8.2) | 2 (4.3) |  |
| 6 | 157 (42.9) | 155 (48.9) | 2 (4.1) |  | 155 (48.6) | 2 (4.3) |  |
| Unknown | 62 (16.9) | 53 (16.7) | 9 (18.4) |  | 54 (16.9) | 8 (17.0) |  |
|  |  |  |  |  |  |  |  |
| **Head abbreviated injury severity score (AIS)** |  |  |  | **0.001** |  |  | **0.001** |
| 1 | 9 (2.5) | 9 (2.8) | 0 (0.0) |  | 9 (2.8) | 0 (0.0) |  |
| 2 | 70 (19.1) | 69 (21.8) | 1 (2.0) |  | 70 (21.9) | 0 (0.0) |  |
| 3 | 112 (30.6) | 109 (34.4) | 3 (6.1) |  | 109 (34.2) | 3 (6.4) |  |
| 4 | 100 (27.3) | 92 (29.0) | 8 (16.3) |  | 91 (28.5) | 9 (19.2) |  |
| 5 | 54 (14.8) | 38 (12.0) | 16 (32.7) |  | 40 (12.5) | 14 (29.8) |  |
| 6 | 21 (5.8) | 0 (0.0) | 21 (42.9) |  | 0 (0.0) | 21 (44.7) |  |
|  |  |  |  |  |  |  |  |
| **Injury severity score** mean[SD]*** | 15.1[10.1] | 14.1[9.3] | 25.3[12.7] | **0.003** | 14.2[9.3] | 25.5[13.0] | 0.003 |
|  |  |  |  |  |  |  |  |
| **Non-head MAXAIS** |  |  |  | **<0.01** |  |  | **<0.01** |
| 0 | 229 (62.6) | 214 (67.5) | 15 (30.6) |  | 214 (67.1) | 15 (31.9) |  |
| 1 | 41 (11.2) | 36 (11.4) | 5 (10.2) |  | 37 (11.6) | 4 (8.5) |  |
| 2 | 34 (9.3) | 30 (9.5) | 4 (8.2) |  | 31 (9.7) | 3 (6.4) |  |
| 3 | 32 (8.7) | 28 (8.8) | 4 (8.2) |  | 28 (8.8) | 4 (8.5) |  |
| 4 | 5 (1.4) | 5 (1.6) | 0 (0.0) |  | 5 (1.6) | 0 (0.0) |  |
| 5 | 4 (1.1) | 3 (1.0) | 1 (2.0) |  | 3 (0.9) | 1 (2.1) |  |
| 6 | 1 (0.3) | 1 (0.3) | 0 (2.0) |  | 1 (0.3) | 0 (0.0) |  |
| Missing | 20 (5.5) | 0 (0.0) | 20 (40.8) |  | 0 (0.0) | 20 (42.6) |  |
|  |  |  |  |  |  |  |  |
| **Hospital stay (days)******* |  |  |  | 0.07 |  |  | 0.07 |
| mean[SD] | 11.3[14.1] | 9.6[10.1] | 22.1[26.6] |  | 9.6[10.1] | 22.3[27.2] |  |
|  |  |  |  |  |  |  |  |
| **Extracranial injury** |  |  |  | **<0.001** |  |  | **<0.001** |
| No | 329 (90.0) | 302 (95.3) | 27 (55.1) |  | 304 (95.3) | 25 (53.2) |  |
| Yes | 16 (4.4) | 14 (4.4) | 2 (4.1) |  | 14 (4.4) | 2 (4.3) |  |
| Missing | 21 (5.7) | 1 (0.3) | 20 (40.8) |  | 1 (0.3) | 20 (42.6) |  |
|  |  |  |  |  |  |  |  |
| **Any surgery** |  |  |  | 0.7 |  |  | 0.7 |
| No | 258 (70.5) | 222 (70.0) | 36 (73.5) |  | 224 (70.2) | 34 (72.3) |  |
| Yes | 108 (29.5) | 95 (30.0) | 13 (26.5) |  | 95 (29.8) | 13 (27.7) |  |
|  |  |  |  |  |  |  |  |
| **Decompressive craniectomy** |  |  |  | **0.01** |  |  | **0.01** |
| No | 344 (94.0) | 305 (96.2) | 39 (79.6) |  | 306 (95.9) | 38 (80.9) |  |
| Yes | 19 (5.2) | 9 (2.8) | 10 (20.4) |  | 10 (3.1) | 9 (19.2) |  |
| NA / Missing | 3 (0.8) | 3 (1.0) | 0 (0.0) |  | 3 (0.9) | 0 (0.0) |  |
|  |  |  |  |  |  |  |  |

p values are corrected by adjusting clustering effect within trauma centers

*Dichotomous PCPC (favorable outcome = normal, mild-moderate disability vs. poor outcome= severe-vegetative and death)

**Dichotomous POPC (favorable outcome = good-moderate overall performance vs. poor outcome = severe-vegetative state and death)

*****Among 366 study sample, 20 patients died at admission had missing injury severity score (Center1 [N=71]; Center2 [N=85]; Center3 [N=21]; Center4 [N=79]; Center5 [N=41]; Center6 [N=25]; Center7 [N=24])

*****Among 366 study sample, 20 patients died at admission (their hospital LOS was assumed to be 1 day), and LOS could not be calculated for 1 patient due to missing discharge date (Center1 [N=74]; Center2 [N=94]; Center3 [N=22]; Center4 [N=80]; Center5 [N=43]; Center6 [N=25]; Center7 [N=27])
