# Supplementary material for: Benchmarking Prehospital and Emergency Department Care for Argentine Children with Traumatic Brain Injury: For the South American Guideline Adherence Group
Source: PLoS One. 2016 Dec 22;11(12):e0166478. doi: 10.1371/journal.pone.0166478 (PMC5179077; doi:10.1371/journal.pone.0166478)
Supplement: S2 Table — p values are corrected by adjusting clustering effect within trauma centers *Dichotomous PCPC (favorable outcome = normal, mild-moderate disability vs. poor outcome = severe-vegetative and death) **Dichotomous POPC (favorable outcome = good-moderate overall performance vs. poor outcome = severe-vegetative state and death) ***Transportation type has four categories. Private Vehicle combines “Private vehicle” and “Taxi”; Helicopter includes “Helicopter” only; Ambulance combines “Ambulance (doctor)”, “Police”, and “Firefighter’; Other/Unknown combines “Other” and “Unknown **** Among 366 study sample, 27 patients had missing transport time (Center1 [N = 70]; Center2 [N = 80]; Center3 [N = 19]; Center4 [N = 79]; Center5 [N = 41]; Center6 [N = 24]; Center7 [N = 26]) (DOCX) [file pone.0166478.s002.docx]

**S2 Table: Transportation Characteristics of 366 Children with Trauma Brain Injury across Seven Study Centers by Discharge Outcomes (Univariate Associations).**

|  | **Total** | ***Favorable PCPC**** | ***Poor PCPC**** | ***P-value*** | ***Favorable POPC***** | ***Poor POPC***** | ***P-value*** |
| --- | --- | --- | --- | --- | --- | --- | --- |
|  | ***n=366*** | ***n=317*** | ***n=49*** |  | ***n=319*** | ***n=47*** |  |
|  | **N(%)** | ***N(%)*** | ***N(%)*** |  | ***N(%)*** | ***N(%)*** |  |
| **Transportation type***** |  |  |  | **0.02** |  |  | **0.02** |
| Private vehicle | 166 (45.4) | 153 (48.3) | 13 (26.5) |  | 153 (48.0) | 13 (27.7) |  |
| Helicopter | 5 (1.4) | 2 (0.6) | 3 (6.1) |  | 2 (0.6) | 3 (6.4) |  |
| Ambulance | 110 (30.1) | 92 (29.0) | 18 (36.7) |  | 94 (29.5) | 16 (34.0) |  |
| Other/Unknown | 85 (23.2) | 70 (22.1) | 15 (30.6) |  | 70 (21.9) | 15 (31.9) |  |
|  |  |  |  |  |  |  |  |
| **Direct transfer from scene** |  |  |  | 0.26 |  |  | 0.26 |
| Yes | 96 (26.2) | 85 (26.8) | 11 (22.5) |  | 85 (26.7) | 11 (23.4) |  |
| No | 267 (73.0) | 229 (72.2) | 38 (77.6) |  | 231 (72.4) | 36 (76.6) |  |
| Unknown | 3 (0.8) | 3 (1.0) | 0 (0.0) |  | 3 (0.9) | 0 (0.0) |  |
|  |  |  |  |  |  |  |  |
| **Transport time from scene to study hospitals (hours)****** |  |  |  | 0.08 |  |  | 0.08 |
| mean[SD] | 5.5[6.3] | 5.6[6.5] | 4.6[3.7] |  | 5.6[6.5] | 4.6[3.8] |  |
|  |  |  |  |  |  |  |  |

p values are corrected by adjusting clustering effect within trauma centers

*Dichotomous PCPC (favorable outcome = normal, mild-moderate disability vs. poor outcome= severe-vegetative and death)

**Dichotomous POPC (favorable outcome = good-moderate overall performance vs. poor outcome = severe-vegetative state and death)

***Transportation type has four categories. Private Vehicle combines “Private vehicle” and “Taxi”; Helicopter includes “Helicopter” only; Ambulance combines “Ambulance (doctor)”, “Police”, and “Firefighter’; Other/Unknown combines “Other” and “Unknown

**** Among 366 study sample, 27 patients had missing transport time (Center1 [N=70]; Center2 [N=80]; Center3 [N=19]; Center4 [N=79]; Center5 [N=41]; Center6 [N=24]; Center7 [N=26])
